# *Bulletin* comment: Smoking, mental ill health and electronic cigarettes†

**DOI:** 10.1192/pb.bp.114.048934

**Published:** 2014-10

Despite bans in virtually all public spaces, smoking continues to be an acceptable facet of psychiatric hospital life. The cigarette and a light are used as currency to encourage good behaviour and adherence with the ward regime, making staff complicit in the supremely health-damaging activity. However, recent evidence contradicts many previous concerns about implementing an immediate blanket ban on smoking anywhere in psychiatric hospitals and their grounds. So why the slow uptake? The reluctance to act is mired in a number of (false) institutional beliefs: that smoking promotes a safe, calm environment, that it is the final pleasure in the otherwise joyless life of a mental health patient, or perhaps it persists because staff themselves smoke.

Condoning smoking reinforces the stigmatisation of psychiatric illness, patients and the facilities treating them. Whereas the rest of society is continually encouraged to quit on health grounds, the psychiatric hospital stands out as a beacon of hypocritical liberalism. Such a situation is anachronistic, belonging to the era of ‘One Flew Over the Cuckoo’s Nest’ when patients demanded their fags from the tyrant Nurse Ratched. This is not to say that there would not be resistance to a ban, as addiction is (by definition) difficult to overcome. The advent of electronic cigarettes, however, provides a real opportunity for psychiatric hospitals to go smoke free. A prescription of regular vaping for dyed-in-the-wool smokers could be an important addition to the armamentarium of those committed to changing the status quo.

Difficult decisions require strong leadership but without a clear message to all hospitals - acute and mental health trusts alike - health inequalities will remain: people with serious psychiatric disorders die 15-20 years earlier than the general population and the cause is mainly cardiovascular and respiratory disorders. Whereas 75% of people with schizophrenia smoke the comparable figure for the general population is 21%. Smoking in mental health facilities should be banned immediately.

